# Health of Cities

**Published:** 1861-01

**Authors:** 


**HEALTH OF CITIES.**—Estimating New-York to contain a million of people, and London two millions and a half, five hundred and fifty dying every week on an average in New-York and twelve hundred and twenty-five in London, the mortality of the two great commercial centers of the Old and the New World is just the same. But there is no city in the civilized world so admirably adapted for health as New-York, bounded as it is on two of its three sides by large rivers, while its topical formation is such that it has an almost perfect drain from its longitudinal center to the rivers. A proper attention to two things would make it exceed any great city in healthfulness. First, a more perfect system of street-cleaning. Second, a better class of dwellings for the poor, combining perfect ventilation and cleanliness.

